# Associação entre Fatores de Risco Cardiovascular e Placas nas Artérias Carótidas em um Estudo Populacional – Estudo SHIP-Brasil

**DOI:** 10.36660/abc.20240546

**Published:** 2025-04-16

**Authors:** Fernanda Burger Zimmermann, Sérgio Luiz Zimmermann, Marcelo Burger Zimmermann, Enrico Klug Beraldi, Mateus Campanelli Franco da Rocha, Bruno Pereira de Oliveira Zabot, Siegmar Starke, Ernani Tiaraju de Santa Helena, Marcello Ricardo Paulista Markus

**Affiliations:** 1 Fundação Universidade Regional de Blumenau Blumenau SC Brasil Fundação Universidade Regional de Blumenau (FURB), Blumenau, SC – Brasil; 2 Hospital Santa Isabel Blumenau Blumenau SC Brasil Hospital Santa Isabel Blumenau, Blumenau, SC – Brasil; 3 University Medicine Greifswald Department and Polyclinic of Internal Medicine B Greifswald Mecklenburg-Vorpommern Alemanha University Medicine Greifswald Department and Polyclinic of Internal Medicine B, Greifswald, Mecklenburg-Vorpommern – Alemanha; 4 German Centre for Cardiovascular Research Greifswald Alemanha German Centre for Cardiovascular Research, Greifswald – Alemanha; 5 German Center for Diabetes Research Greifswald Alemanha German Center for Diabetes Research, Greifswald – Alemanha

**Keywords:** Aterosclerose, Espessura Intima-Media Carotídea, Fatores de Risco de Doenças Cardíacas, Ultrassonografia das Artérias Carótidas

## Abstract

**Fundamento:**

A doença aterosclerótica é uma causa relevante de morbidade e mortalidade na população geral, e importante para detectar fatores que possam influenciar sua prevenção.

**Objetivos:**

Avaliar a associação de fatores de risco cardiovascular com placas na artéria carótida em participantes do Estudo de Saúde na Pomerânia (*The Study of Health in Pomerania*, SHIP).

**Métodos:**

Um total de 1953 participantes foram avaliados quanto à presença de fatores de risco cardiovasculares (hipertensão, dislipidemia, diabetes tipo 2, obesidade, tabagismo e inatividade física) e variáveis sociodemográficas (sexo, idade, cultura alemã, cor/raça autodeclarada, e consumo de álcool), circunferência da cintura, relação cintura/quadril, relação cintura-altura. A presença de placas na artéria carótida foi analisada por ultrassonografia. A associação entre as variáveis do estudo e a presença de placas foi avaliada pelo teste do qui-quadrado. Um valor de p<0,05 foi considerado significativo.

**Resultados:**

As placas estiveram presentes em 56,5% dos pacientes hipertensos (p<0,001), 49,8% dos pacientes dislipidêmicos (p<0,001), 62% dos pacientes diabéticos (p<0,001), 52% dos fumantes e 29% dos pacientes que nunca fumaram (p<0,001), 39,5% dos indivíduos sedentários e 33,1% dos não sedentários (p=0,014), 43,7% das pessoas obesas e 26,1% dos eutróficos (p<0,001).

**Conclusão:**

Placas nas artérias carótidas foram prevalentes em indivíduos do sexo masculino, com idade entre 60 e 79 anos, da raça branca, hipertensos, dislipidêmicos, diabéticos, tabagistas, sedentários e obesos, de cultura alemã, analfabetos, da classe econômica A1/A2 e com baixo consumo de álcool.

## Graphical abstract

**Figura Central:**
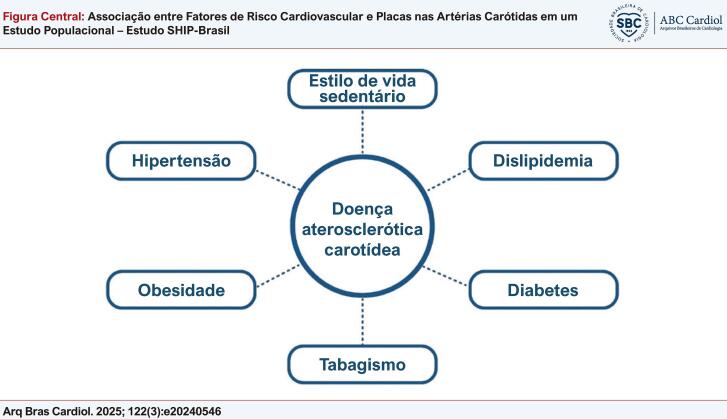
: Associação entre Fatores de Risco Cardiovascular e Placas nas Artérias Carótidas em um Estudo Populacional – Estudo SHIP-Brasil

## Introdução

As doenças cardiovasculares ateroscleróticas estão associadas a altas taxas de morbidade e mortalidade em todo o mundo.^1,2^ A aterosclerose é uma doença inflamatória crônica causada por um desequilíbrio no metabolismo lipídico, principalmente devido à elevada concentração de lipoproteína de baixa densidade (LDL colesterol) no sangue, gerando placas ateromatosas nos vasos.^3,4^ Os principais fatores de risco para o desenvolvimento da doença são hipertensão, dislipidemia, diabetes, tabagismo, obesidade, estilo de vida sedentário e histórico familiar, além da influência de fatores como idade e sexo.^5,6^

As primeiras manifestações clínicas das doenças cardiovasculares ateroscleróticas incluem angina estável, angina instável e sinais de doença cardíaca isquêmica.^7^ Elas também podem ser silenciosas e se manifestar como um evento potencialmente fatal como o infarto agudo do miocárdio. Diante disso, a estratificação de risco é extremamente importante para identificar indivíduos assintomáticos com uma maior predisposição para desenvolverem doença cardiovascular.^8^

Além dos vários escores de estratificação de risco existentes, é possível identificar a presença de placas ateroscleróticas nas artérias carótidas. Trata-se de um marcador subclínico de doença aterosclerótica que pode ser avaliado pelo exame de ultrassonografia.^9^ As Diretrizes Brasileiras de Dislipidemia classificaram a aterosclerose subclínica, detectada pela presença de placas nas artérias carótidas, como um indicador de alto risco para o desenvolvimento de doença cardiovascular em indivíduos submetidos à prevenção primária.^10,11^ A investigação desses marcadores é de grande importância.

Nesse contexto, nosso estudo teve como objetivo avaliar a associação de fatores de risco cardiovascular com placas nas artérias carótidas. Isso é importante, uma vez que a identificação dos grupos de pacientes com maior risco de desenvolverem doença aterosclerótica carotídea pode levar a melhores programas de rastreamento em sistemas de saúde.

## Métodos

Este é um estudo epidemiológico, transversal, de base populacional de participantes no momento basal do Estudo de Saúde na Pomerânia (*The Study of Health in Pomerania*, SHIP).

O SHIP-Brazil é um estudo do tipo coorte conduzido por pesquisadores do Programa de Graduação em Saúde Pública da Fundação Universidade Regional de Blumenau (FURB) em parceria com o Instituto de Medicina de Comunidade da Faculdade de Medicina de Greifswald, Universidade de Greifswald Alemanha. Os objetivos, delineamento, métodos e resultados preliminares do estudo foram descritos previamente.^12^

Em resumo, obteve-se uma amostra aleatória simples, com pessoas com idade entre 20 e 79 anos de ambos os sexos (12 estratos de amostragem, dois estratos por sexo e seis estratos por grupo etário de dez anos), com um total de 3678 participantes. O cálculo amostral considerou uma prevalência de eventos de 50%, precisão de 5% e intervalo de confiança de 95%. Os critérios de inclusão foram pessoas que residiam em Pomerode, Santa Catarina, por pelo menos seis meses. Os critérios de exclusão foram pacientes com alguma condição física e/ou mental que os impediram de responder os questionários ou ser submetidos a exames. Ao final da coleta de dados, no estudo, ocorreram cerca de 30% de perdas e/ou recusas, e a amostra final foi composta de 2488 pessoas.

A coleta de dados foi realizada entre 2014 e 2016. Os participantes receberam visita no domicílio, onde os questionários foram administrados. Ao final da visita, os participantes receberam as orientações necessárias para realizarem exames no hospital universitário de Blumenau. Todos os procedimentos de coleta de dados estão descritos nos Procedimentos Operacionais Padrão (http://www.furb.br/vspomerode).

Os exames de ultrassonografia das artérias carótidas foram realizados por um examinador treinado, usando o equipamento Vivid^TM^ I (General Electric), equipado com um transdutor e um sistema de registro de imagem. Os participantes permaneceram na posição supina com a cabeça ligeiramente inclinada para o lado contralateral da artéria carótida. Cada segmento – artéria carótida comum distal, bifurcação da carótida e as porções proximais da artéria carótida interna e da artéria carótida externa à direita e à esquerda – foi examinado separadamente e em sequência. Primeiramente, a imagem em modo B foi estudada tanto longitudinalmente e em uma imagem transversal em relação às placas ateroscleróticas. Aqui, os vasos foram examinados com diferentes ângulos de sons. Isso significa que, do ângulo localizado na posição mais anterior até o ângulo localizado na posição mais posterior, todos os vasos devem ser examinados para detectar até as menores placas excêntricas. A presença de placa foi considerada quando houvesse (1) uma protrusão focal do complexo médio-intimal em direção ao lúmen do vaso, que emergisse acima da espessura da camada da íntima-media circundante; ou (2) um aumento focal na ecogenicidade em uma seção longitudinal determinada por uma aspereza da superfície luminal, mesmo sem projeção ou com uma mínima projeção para o lúmen do vaso. As placas foram avaliadas visualmente e quantificadas com base na localização.

As seguintes variáveis sociodemográficas foram escolhidas: sexo (masculino/feminino), grupo etário: 20 a 39, 40 a 59 e 60 a 79, estado civil – casado e solteiro(a)/separado(a)/viúvo(a), raça/cor autodeclarada e educação (analfabeto, ensino fundamental, ensino médio, faculdade). Os indivíduos que relataram falar alemão regularmente em casa e frequentar associações culturais alemãs foram considerados de cultura alemã.^13^ A classe econômica de consumo foi obtida de acordo com o “Critério Brasil”,^14^ e a soma dos escores foi convertida em categorias que foram agrupadas em A1/A2, B1/B2, C1/C2, ou D/E.

Quanto às variáveis de estilo de vida, os participantes foram classificados em fumantes, ex-fumantes e não fumantes. O consumo de bebida alcoólica (baixo, moderado, alto e grave) foi estimado usando o questionário AUDIT-C adaptado e validado em português.^15^ O *International Physical Activity Questionnaire* (IPAQ-versão curta)^15,16^ foi usado para estimar a atividade física, e os que relataram praticar atividade física moderada ou vigorosa por 150 minutos ou mais por semana foram considerados suficientemente ativos.

As variáveis antropométricas incluíram índice de massa corporal (IMC), razão cintura-quadril (RCQ), razão cintura-altura (RCA) e circunferência da cintura (CC). A massa corporal foi medida usando uma balança eletrônica (WELMY^®^ Adult W300) com capacidade de 300 Kg e precisão de 50g. A altura foi medida usando um estadiômetro com uma haste fixa à balança. O IMC foi obtido dividindo-se a massa corporal (em quilogramas) pela altura ao quadrado (m^2^). Os participantes foram classificados como eutróficos (IMC < 25 kg/m^2^), com sobrepeso (25,0 a 29,9 Kg/m^2^) e obesos (≥ 30 Kg/m^2^). Os idosos foram considerados com sobrepeso se apresentassem IMC ≥ 27 Kg/m^2^). As circunferências foram medidas usando uma fita inelástica Cescorf^®^ (em centímetros). A RCQ foi calculada, e os valores menores que 0,85 para mulheres e menores que 0,90 para homens foram considerados inadequados.

A presença de hipertensão arterial, Diabetes Mellitus e dislipidemia foi autorrelatada pelos participantes com base em um diagnóstico clínico prévio.

### Análise estatística

Os dados foram analisados usando estatística descritiva – média e desvio padrão (DP) e/ou frequências (absolutas e relativas) e apresentados em tabelas. As variáveis foram testadas quanto à distribuição (assimetria e curtose usando o teste de D’Agostino). A associação entre as variáveis do estudo e o escore da placa (nenhum, um ou dois, e três ou mais)^17^ foi examinada usando o programa Stata 11,2 (Stata Corporation, College Station, TX, EUA). Um valor de p <0,05 foi considerado significativo.

O SHIP-Brazil segue a resolução 466/2012 e a resolução 510/2016 do Conselho Nacional da Saúde, bem como a Declaração de Helsinki. Todos os participantes assinaram o termo de consentimento, e o estudo foi submetido e aprovado ao comitê de ética da FURB (2.969.842).

## Resultados

A população do estudo foi composta de 2488 participantes. Desses, 235 (9,4%) não compareceram ao centro de exames e 300 (12,1%) não aceitaram a serem submetidos ao exame de ultrassonografia das carótidas. Nós analisamos 1953 participantes.

A Tabela 1 apresenta as características dos participantes. A idade média foi 50,85 anos (DP=14.64) e a mediana foi 51,4 anos. Observou-se uma prevalência mais alta de participantes do sexo feminino, estado civil casado, raça branca e cultura alemã. Quanto à escolaridade, a maioria dos participantes haviam completado entre um e quatro anos de ensino fundamental. A classe B1/B2 foi a predominante. Em relação ao estilo de vida, observou-se um baixo consumo de álcool na população do estudo, bem como uma alta porcentagem de pessoas que nunca haviam fumado, e uma baixa porcentagem de pessoas sedentárias. Em relação aos dados antropométricos, houve uma alta prevalência de obesidade, CC de alto risco, RCQ de alto risco, e RCQ de alto risco. Foi possível observar ainda uma porcentagem baixa de participantes com doenças crônicas: hipertensão, diabetes e dislipidemia em comparação aos participantes sem doenças crônicas. Neste estudo, a presença de placas nas artérias carótidas foi observada em 36% dos participantes: 64,0% não apresentaram placas, 23,3% apresentaram uma ou duas placas e 12,8% apresentaram três ou mais placas nas artérias carótidas.

**Tabela 1:** – Características dos participantes do “The Study of Health in Pomerania (SHIP) – Brazil” 2014-2018

Variáveis		Características da população (n = 1953)	
		Frequência	Porcentagem
**1 – Sexo**	Masculino	820	42,0%
	Feminino	1133	58,0%
**2 – Idade**	20-39	498	25,5%
	40-59	885	45,3%
	60-79	570	29,2%
**3 – Estado civil**	Casado	1482	77,2%
	Solteiro/Separado/Viúvo	438	22,8%
**4 – Raça/Cor**	Branca	1779	92,8%
	Não-branca	138	7,2%
**5 – Cultura alemã**	Não	572	29,8%
	Sim	1345	70,2%
**6 – Educação**	Ensino superior	263	13,9%
	Ensino médio	493	26,0%
	Ensino fundamental (5º - 8º ano)	374	19,8%
	Ensino fundamental (1º - 4º ano)	739	39,0%
	Analfabeto	24	1,3%
**7 – Classe socioeconômica**	A1/A2	244	12,5%
	B1/B2	1148	58,8%
	C1/C2	547	28,0%
	D/E	13	0,7%
**8 – Consumo de álcool**	Baixo	1346	70,8%
	Moderado	322	16,9%
	Alto	150	7,9%
	Grave	83	4,4%
**9 – Tabagismo**	Nunca fumou	1271	66,7%
	Tabagista	434	22,8%
	Ex-tabagista	202	10,6%
**10 – Atividade física**	Ativo	1239	68,3%
	Sedentário	575	31,7%
**11 – Obesidade**	Baixo peso	15	0,8%
	Eutrófico	445	24,2%
	Sobrepeso	668	36,3%
	Obeso	712	38,7%
**12 – Circunferência da cintura**	Risco baixo	632	34,6%
	Risco alto	1194	65,4%
**13 – Relação cintura-altura**	Risco baixo	558	30,5%
	Risco alto	1269	69,5%
**14 – Relação cintura-altura**	Risco baixo	551	30,1%
	Risco alto	1277	69,9%
**15 – Hipertensão**	No	1100	63,2%
	Yes	640	36,8%
**16 – Diabetes**	No	1719	91,0%
	Yes	171	9,0%
**17 – Dislipidemia**	No	1297	68,2%
	Yes	604	31,8%
**18 – Placas na artéria carótida**	0	1249	64,0%
	1-2	455	23,3%
	≥3	249	12,8%

Fonte: The Study of Health in Pomerania (SHIP) – Brazil

As Tabelas 2 e 3 apresentam a associação entre as variáveis apresentadas e a presença de placas ateroscleróticas na artéria carótida. Observou-se uma porcentagem mais alta de participantes sem placas com as seguintes características: sexo feminino, idade entre 20 e 39 anos, solteira/separada/viúva, raça não branca, cultura não alemã, educação superior completa, classe econômica B1/B2, consumo moderado de álcool, nunca fumaram, e praticava atividade física. Quanto aos dados antropométricos, a porcentagem mais alta de pessoas sem placas encontrava-se no grupo eutrófico, com medidas de CC, RCQ e RCA de baixo risco. Quanto às doenças crônicas, havia uma porcentagem mais alta de indivíduos sem placas naqueles não hipertensos, não dislipidêmicos e não diabéticos.

**Tabela 2:** – Associação entre variáveis sociodemográficas e de estilo de vida e risco cardiovascular com a presença de placas na artéria carótida em participantes do estudo “The Study of Health in Pomerania (SHIP) – Brazil” 2014-2018

Características		Escore de placas			Valor p
		0 n = 1249	1 ou 2 n = 455	≥3 n = 249	
**1 – Estado civil**	Casado	948 (64%)	350 (23,6%)	184 (12,4%)	0,690
	Solteiro / Divorciado / Viúvo	290 (66,2%)	97 (22,2%)	51 (11,6%)	
**2 – Cultura alemã**	Não	458 (80,1%)	84 (14,7%)	30 (5,2%)	<0,001
	Sim	778 (57,8%)	362 (26,9%)	205 (15,2%)	
**3 – Educação**	Ensino superior	231 (87,8%)	25 (9,5%)	7 (2,7%)	<0,001
	Ensino médio	407 (82,6%)	70 (14,2%)	16 (13,7%)	
	Ensino fundamental (5º - 8º ano)	261 (69,8%)	77 (20,6%)	36 (9,6%)	
	Ensino fundamental (1º - 4º ano)	310 (42,0%)	252 (34,1%)	177 (24,0%)	
	Analfabeto	10 (41,7%)	11 (45,8%)	3 (12,5%)	
**4 – Classe socioeconômica**	A1/A2	137 (56,2%)	66 (27,0%)	41 (16,8%)	<0,001
	B1/B2	784 (68,3%)	251 (21,9%)	113 (9,8%)	
	C1/C2	320 (58,5%)	136 (24,9%)	91 (16,6%)	
	D/E	8 (61,5%)	1 (7,7%)	4 (30,8%)	
**5 – Consumo de álcool**	Baixo	828 (61,5%)	337 (25,0%)	181 (13,5%)	0,001
	Moderado	239 (74,2%)	57 (17,7%)	26 (8,1%)	
	Alto	107 (71,3%)	28 (18,7%)	15 (10,0%)	
	Grave	55 (66,3%)	18 (21,7%)	10 (12,0%)	
**6 – Tabagismo**	Nunca fumou	903 (71,0%)	263 (20,7%)	105 (8,3%)	<0,001
	Tabagista	235 (54,2%)	118 (27,2%)	81 (18,7%)	
	Ex-tabagista	97 (48,0%)	59 (29,2%)	46 (22,8%)	
**7 – Atividade física**	Ativo	829 (66,9%)	263 (21,1%)	147 (11,9%)	0,014
	Sedentário	348 (60.5%)	156 (27.1%)	71 (12.4%)	

Fonte: The Study of Health in Pomerania (SHIP) – Brazil

**Tabela 3:** – Associação de variáveis clínicas e antropométricas e risco cardiovascular com a presença de placas na artéria carótida com a presença de placas na artéria carótida nos participantes do estudo “The Study of Health in Pomerania (SHIP) – Brazil” 2014-2018

Características		Escore de placas			Valor p
		0 n = 1249	1 ou 2 n = 455	≥3 n = 249	
**1 – Sexo**	Masculino	481 (58,7%)	199 (24,3%)	140 (17%)	<0,001
	Feminino	768 (67,8%)	256 (22,6%)	109 (9,6%)	
**2 – Grupo etário**	20-39	483 (97%)	14 (2,8%)	1 (0,2%)	<0,001
	40-59	623 (70,4%)	206 (23,3%)	56 (6,3%)	
	60-79	143 (25,1%)	235 (41,2%)	192 (33,7%)	
**3 – Raça / Cor**	Branca	1126 (63,3%)	426 (24%)	227 (12,8%)	<0,001
	Não branca	111 (80,4%)	21 (15,2%)	6 (4,4%)	
**4 – Obesidade**	Baixo peso	11 (73,3%)	0 (0%)	4 (26,7%)	<0,001
	Eutrófico	329 (73,9%)	68 (15,3%)	48 (10,8%)	
	Sobrepeso	442 (66,2%)	151 (22,6%)	75 (11,2%)	
	Obeso	401 (56,3%)	209 (29,4%)	102 (14,3%)	
**5 – Circunferência abdominal**	Risco baixo	465 (73,6%)	99 (15,7%)	68 (10,8%)	<0,001
	Risco alto	713 (59,7%)	326 (27,3%)	155 (13,0%)	
**6 – Relação cintura-altura**	Risco baixo	424 (76,0%)	79 (14,2%)	55 (9,9%)	<0,001
	Risco alto	754 (59,4%)	347 (27,3%)	168 (13,2%)	
**7 – Relação cintura-quadril**	Risco baixo	467 (84,8%)	57 (10,3%)	27 (4,9%)	<0,001
	Risco alto	712 (55,8%)	369 (28,9%)	196 (15,4%)	
**8 – Hipertensão**	Não	845 (76,8%)	188 (17,1%)	67 (6,1%)	<0,001
	Sim	279 (43,6%)	216 (33,8%)	145 (22,7%)	
**9 – Diabetes**	Não	1160 (67,5%)	381 (22,2%)	178 (10,4%)	<0,001
	Sim	65 (38,0%)	56 (32,8%)	50 (29,2%)	
**10 – Dislipidemia**	Não	923 (71,2%)	256 (19,7%)	118 (9,1%)	<0,001
	Sim	303 (50,2%)	185 (30,6%)	116 (19,2%)	

Fonte: The Study of Health in Pomerania (SHIP) – Brazil

Uma porcentagem mais alta de participantes com uma ou duas placas ateroscleróticas foi encontrada com as seguintes características: sexo masculino, idade entre 60 e 79 anos, casado, branco, cultura alemã, analfabeto, classe econômica A1/A2, baixo consumo de álcool, fumantes, insuficientemente ativos, hipertenso, diabético e dislipidêmico. Quanto aos dados antropométricos, houve uma porcentagem mais alta de obesidade, medida de CC de alto risco, medida de RCA de alto risco e RCQ de baixo risco.

Observou-se uma porcentagem mais alta de participantes com três ou mais placas entre os indivíduos do sexo masculino, idade entre 60 e 79 anos, raça branca, cultura alemã, analfabetos, classe econômica A1/A2, baixo consumo de álcool, tabagistas, sedentários, obesos, hipertensos, diabéticos e dislipidêmicos.

## Discussão

A presença de aterosclerose carotídea trem sido descrita em uma parcela considerável de populações na América Latina há décadas. Um estudo mexicano publicado em 1999 relatou uma prevalência de lesão aterosclerótica carotídea em 64,8% da população estudada.^18^ Um estudo brasileiro^19^ publicado em 2021 demonstrou uma frequência de presença de placas em 35,8% da população.

Em relação ao gênero, encontramos que a prevalência de pessoas com placas na artéria carótida foi mais alta entre homens. Isso foi observado em participantes com uma ou duas placas e naqueles com três ou mais placas. Essa informação está de acordo com achados de estudos prévios^8,20,21^ que demonstram que homens estão em um risco maior de eventos cardiovasculares. Por outro lado, em mulheres após a menopausa, esse risco é igualado pela ausência de efeito protetor proporcionado pelo estrógeno.^22-24^

Além disso, observou-se que à medida que o grupo etário aumentava, ocorria um aumento na prevalência dos participantes com placas na artéria carótida. Vale mencionar que a porcentagem dos participantes com placas na artéria carótida no grupo 40-59 anos foi significativamente maior que a porcentagem encontrada no grupo 20-39 anos. Ainda, a porcentagem dos participantes com placas na artéria carótida no grupo 60-79 anos aumentou de 29,6% para 74,9% em comparação ao grupo etário anterior. Essa característica foi descrita em outros estudos, que observou um aumento substancial nas placas nos participantes com idade de 50 anos ou mais.^20,21,25,26^

A hipertensão é um importante fator de risco para o desenvolvimento de placas ateroscleróticas, como demonstrado em vários estudos.^8,21,22,25-30^ Da mesma forma, o diabetes é caracterizado em várias fontes de literatura como uma importante comorbidade associada à formação de placa.^20,21,25,26^

Em nosso estudo, encontrou-se uma forte correlação entre dislipidemia e a presença de placas. No grupo dislipidêmico, placas ateroscleróticas estiveram presentes em mais da metade dos participantes. Essa correlação também foi observada em vários estudos em que essa variável foi analisada.^21,25,26^

Em relação ao estilo de vida, as pessoas sedentárias apresentaram uma prevalência significativamente mais alta de placas na artéria carótida que em pessoas fisicamente ativas (p=0,014), e essa associação também foi observada em outros estudos.^21,22,25,26^ Em relação ao tabagismo, encontrou-se uma porcentagem mais alta de placas em tabagistas atuais, mas também foi observada uma alta prevalência de placas em ex-tabagistas. Estudos sugerem que a taxa de alteração aterosclerótica pode ser reduzida por interrupção do tabagismo, mas um efeito residual parece estar presente por pelo menos 10 anos.^31,32^ A cronologia desses fatos pode não ser explicada pelo processo de inflamação crônica associado com tabagismo, o que pode danificar células endoteliais das artérias carótidas e favorecer o acúmulo de fatores trombogênicos.^33^

Quanto ao consumo de álcool, houve uma alta prevalência de placas em pessoas com baixo consumo de álcool. Outro estudo baseado no SHIP utilizou a espessura da camada íntima-média carotídea como um método de avaliar aterosclerose subclínica, e encontrou uma associação inversamente proporcional entre consumo de álcool e um aumento na espessura da camada íntima-média, outro marcador subclínico de aterosclerose. Essa associação foi observada em homens com uma ingestão diária de até 80 g de álcool.^34^

Nossos resultados demonstram uma associação entre obesidade e presença de placas. Uma relação similar também foi observada entre medidas de alto risco da CC, RCA, e RCQ e a presença de placas. Essa observação foi encontrada em vários estudos publicados.^21,25,26^

Em relação à escolaridade, níveis mais baixos foram associados com prevalência mais alta de placas. Essa relação pode ser explicada pelo baixo nível educacional dessa população em relação à prevenção de doenças. Quanto à classe socioeconômica, a porcentagem mais alta de participantes com uma ou duas placas encontrava-se na classe A1/A2 (27,1%), e a prevalência mais alta de três ou mais placas encontrava-se na classe D/E (30,8%). Outros estudos associaram o baixo nível educacional e socioeconômico dos participantes com a presença de aterosclerose subclínica.^26^ Estudos futuros poderão demonstrar se indivíduos de classes sociais mais altas possam ter maior acesso a exames complementares, enquanto em classes sociais mais baixas, o diagnóstico seria feito quando a doença já estivesse avançada.

Quanto à etnia, houve uma prevalência mais baixa de placas carótidas em pessoas da raça branca. Segundo as diretrizes de prevenção da *American Heart Association*, o risco estimado de desenvolver doença cardiovascular aterosclerótica em 10 anos é mais alto em pessoas afro-americanas.^34^Nós observamos uma prevalência mais alta de placas em participantes de cultura alemã (usado aqui como substituto de ascendência alemã). Um estudo avaliando descendentes de alemães na região do Vale do Itajaí mostrou que descendentes da primeira geração de alemães não apresentaram diferenças significativas em termos de fatores de risco cardiovascular em comparação a imigrantes alemães, exceto pelo colesterol HDL, que foi mais alto na população alemã, embora essa fração não tenha sido analisada em nossa população.^35^

Não existem dados na literatura que indicam diferenças significativas no número de placas ateroscleróticas. De fato, não observamos diferenças significativas entre os grupos com 1-2 placas e grupos com três ou mais placas na artéria carótida que pudessem influenciar decisões clínicas.

O estudo de fatores de risco e de características prevalentes na associação com doença aterosclerótica pode levar a medidas de prevenção e de controle para combater seus efeitos deletérios. Mas, é importante mencionar que esse estudo pode ter algumas limitações. Por ser um estudo transversal, ele não define uma relação de causa e efeito entre as variáveis, somente levantando hipóteses que requerem análises novas e mais profundas em estudos longitudinais para confirmá-las. Vale ainda ressaltar o fato de algumas informações clínicas terem sido autorrelatadas pela população em análise, e o alto número de perdas e recusas, que podem gerar vieses. Esse fator pode ser mais bem analisado no futuro por revisões de prontuários médicos e por outros testes confirmatórios.

## Conclusões

Houve uma associação entre a presença de fatores de risco cardiovasculares e a presença de placas na artéria carótida nos participantes. As placas foram mais prevalentes nos indivíduos hipertensos, dislipidêmicos, diabéticos, tabagistas, sedentários e obesos. Quanto às características da população, as placas eram mais prevalentes em homens, com idade entre 60 e 79 anos, de cultura alemã, analfabeto, da classe socioeconômica A1/A2 e com baixo consumo de álcool. As placas ateroscleróticas eram mais prevalentes entre os participantes com valores de alto risco para CC, RCQ e RCA.

Esses achados destacam a importância de se identificar grupos de risco que se beneficiariam mais consistentemente de protocolos de rastreamento com bom custo-benefício, sem serem expostos desnecessariamente a procedimentos invasivos que pudessem levar a danos físicos e psicológicos. Assim, estudos longitudinais e delineamentos experimentais são necessários para melhorar a avaliação de causalidade e progressão de risco.
