# Supplementary material for: Associação entre Fatores de Risco Cardiovascular e Placas nas Artérias Carótidas em um Estudo Populacional – Estudo SHIP-Brasil
Source: Arq Bras Cardiol. 2025 Apr 16;122(3):e20240546. [Article in Portuguese] doi: 10.36660/abc.20240546 (PMC12129473; doi:10.36660/abc.20240546)
Supplement: Translation

# Translation: Association between Cardiovascular Risk Factors and Carotid Plaques in a Population-Based Study - The SHIP-Brazil Study

**DOI:** 10.36660/abc.20240546i

**Authors:** Fernanda Burger Zimmermann, Sérgio Luiz Zimmermann, Marcelo Burger Zimmermann, Enrico Klug Beraldi, Mateus Campanelli Franco da Rocha, Bruno Pereira de Oliveira Zabot, Siegmar Starke, Ernani Tiaraju de Santa Helena, Marcello Ricardo Paulista Markus

**Affiliations:** 1 Brazil Fundação Universidade Regional de Blumenau (FURB), Blumenau, SC – Brazil; 2 Brazil Hospital Santa Isabel Blumenau, Blumenau, SC – Brazil; 3 Germany University Medicine Greifswald Department and Polyclinic of Internal Medicine B, Greifswald, Mecklenburg-Vorpommern – Germany; 4 Germany German Centre for Cardiovascular Research, Greifswald – Germany; 5 Germany German Center for Diabetes Research, Greifswald – Germany

**Keywords:** Atherosclerosis, Carotid Intima-Media Thickness, Heart Disease Risk Factors, Carotid Arteries Ultrasonography

## Abstract

**Background:**

Atherosclerotic disease is a relevant cause of morbidity and mortality in the general population, and it is important to detect factors that can influence its prevention.

**Objective:**

To evaluate the association of cardiovascular risk factors with carotid plaques in participants of the Study of Health in Pomerania (SHIP) – Brazil.

**Methods:**

A total of 1,953 participants were assessed for the presence of cardiovascular risk factors (hypertension, dyslipidemia, type 2 diabetes, obesity, smoking and physical inactivity) and sociodemographic variables (gender, age group, Germanic culture, color/race self-declared, and alcohol consumption), waist circumference measurement, waist-to-hip ratio and waist-to-height ratio. The presence of carotid plaques was analyzed by ultrasound. The association between the study variables and carotid plaques was assessed using the Chi-square test. A p-value < 0.05 was considered significant.

**Results:**

Plaques were present in 56.5% of hypertensive patients (p<0.001), 49.8% of dyslipidemic patients (p<0.001), 62% of diabetic patients (p<0.001), 52% of smokers, 29% of those who had never smoked (p<0.001), 39.5% of sedentary people and 33.1% of non-sedentary people (p=0.014), 43.7% of obese people and 26.1% of eutrophic people (p<0.001).

**Conclusion:**

Carotid plaques were prevalent in men, aged between 60 and 79, white, hypertensive, dyslipidemic, diabetic, smokers, sedentary and obese, illiterate, in economic class A1/A2, and of Germanic culture, and low alcohol consumption.

## Introduction

Atherosclerotic cardiovascular diseases are associated with high rates of morbidity and mortality worldwide.^1,2^ Atherosclerosis is a chronic inflammatory disease caused by an imbalance in lipid metabolism, mainly due to the high concentration of LDL cholesterol in the blood, generating atheromatous plaques in the vessels.^3,4^The main risk factors for developing this disease are hypertension, dyslipidemia, diabetes, smoking, obesity, sedentary lifestyle and family history; in addition to the influence of factors such as age and gender.^5,6^

Atherosclerotic cardiovascular diseases can have as their first clinical manifestation: stable angina, unstable angina or signs of ischemic heart disease.^7^They can also be silent and manifest as a potentially fatal event such as acute myocardial infarction. In view of this, risk stratification is extremely important to identify asymptomatic individuals with a greater predisposition to develop cardiovascular disease.^8^

In addition to numerous risk stratification scores, it is possible to identify the presence of atherosclerotic plaques in the carotid arteries. This is a subclinical marker for atherosclerotic disease that can be assessed using ultrasound.^9^The Brazilian Dyslipidemia Guidelines have classified the finding of subclinical atherosclerosis through the presence of carotid plaques, as a high-risk indicator for the development of cardiovascular disease in individuals undergoing primary prevention.^10,11^The investigation of this marker is of great importance.

In this context, our study sought to evaluate the association of cardiovascular risk factors with carotid plaques. This is important because the identification of groups of patients at higher risk for carotid atherosclerosis may lead to better screening programs in health systems.

## Methods

This is a population-based, cross-sectional epidemiological study of participants in the baseline of the “The Study of Health in Pomerode - SHIP Brazil” study.

SHIP-Brazil is a cohort study conducted by researchers from the Graduate Program in Public Health at the Regional University of Blumenau (FURB) in partnership with the Institute of Community Medicine of the Greifswald Medical School, University of Greifswald, Germany. The objectives, design, methods and preliminary results were described previously.^12^

In short, a simple random sample was drawn, with people aged between 20 and 79 of both sexes (12 sampling strata, two strata by sex and six strata by 10-year age group), totaling 3678 participants. The sample calculation considered a prevalence of events of 50%, a precision of 5% and a 95% confidence interval. Inclusion criteria were people who had lived in Pomerode, Brazil, for at least six months. Exclusion criteria: patients with a physical and/or mental disorder that prevented them from answering the questionnaires or undergoing tests. At the end of data collection, the study had around 30% losses and/or refusals, and the final sample totaled 2488 people.

Data collection took place between 2014 and 2018. Participants were visited at home, where the questionnaires were administered. At the end of the visit, the participants were given the necessary instructions for taking the exams at the Examination Center located at the Blumenau University Hospital (CE-HUB). All data collection procedures are described in the Standard Operating Procedures (http://www.furb.br/vspomerode).

Ultrasound examinations of the carotid arteries were carried out by a trained examiner, using Vivid^TM^ I (General Electric), equipped with a linear ultrasonic transducer and an image recording system. The participants remained in the supine position with their heads slightly inclined to the side contralateral to the carotid artery being studied. Each individual segment – distal common carotid artery, carotid bifurcation and the proximal portions of the internal carotid artery and external carotid artery, on the right and left – was examined separately and in succession. Firstly the B-mode image was studied both longitudinally and in a cross-sectional image with regard to atherosclerotic plaques. Here, vessels were also examined from different sound angles. This means that from the angle in the most anterior position to the one located in the most posterior position, all vessels should be examined to detect even the smallest eccentric plaques. Plaque was considered to be present when there was (1) a focal protrusion of the intima-media complex into the vessel lumen, which rose above the thickness of the surrounding intima-media layer (IMT), or (2) a focal increase in echogenicity in a longitudinal section determined by a roughness of the luminal surface, even without projection or with a minimal projection into the vessel lumen. Plaques were assessed by visual judgment and quantified based on their location.

The following sociodemographic variables were selected: gender (male/female), age group: 20 to 39, 40 to 59 and 60 to 79, marital status (married and single/separated/widowed), self-declared race/color and education (illiterate, elementary, high school, college). Those who reported speaking German regularly at home and attending German cultural associations were of Germanic culture (GC).^13^ The economic class of consumption was obtained according to “Critério Brasil”,^14^ and the sum of the scores was converted into categories that were grouped into A1/A2, B1/B2, C1/C2, or D/E.

As for lifestyle variables, participants were classified as current smokers, ex-smokers and non-smokers. Alcohol consumption (low, moderate, high and severe) was estimated using the AUDIT-C questionnaire adapted and validated in Portuguese.^15^ The International Physical Activity Questionnaire (IPAQ-short form)^15,16^ was used to estimate physical activity, and those who reported practicing moderate or vigorous physical activity for 150 minutes or more per week were considered sufficiently active.

Anthropometric variables included body mass index (BMI), waist-to-hip ratio (WHR), waist-to-height ratio (WtHR) and waist circumference (WC). Body mass was measured on a WELMY^®^ Adult W300 electronic scale with a capacity of 300kg and 50g divisions. Height was measured using a stadiometer with a rod attached to the scale. BMI was obtained by dividing body mass (in kilograms) by the square of height (in meters). Adult participants were classified as eutrophic (BMI < 25 kg/m^2^), overweight (BMI 25.0 to 29.9 Kg/m^2^) and obese (BMI ≥ 30 Kg/m^2^). Among the elderly, they were considered overweight if BMI ≥ 27 kg/m^2^). Circumferences were measured using a Cescorf^®^ inelastic tape measure (in centimeters). The WHR was calculated, and values less than 0.85 for women and less than 0.90 for men were considered adequate.

The presence of systemic arterial hypertension, diabetes mellitus (DM) and dyslipidemia was self-reported by the participants based on a previous medical diagnosis.

### Statistical analysis

The data were examined using descriptive statistics (mean and standard deviation [SD] and/or frequencies [absolute and relative]) and presented in tabular form. The variables were tested for their distribution (skewness and kurtosis using the D’Agostino test). The association between the study variables and the plaque score (none, one or two and three or more)^17^ was examined using the Chi-square test (categorical variables). Data were analyzed using Stata 11.2 (Stata Corporation, College Station, TX, USA). A p- value of <0.05 was accepted as significant.

SHIP-Brazil complies with Resolution 466/2012 and Resolution 510/2016 of the National Health Council, as well as the Declaration of Helsinki. All participants signed the respective Informed Consent, and the study was submitted to and approved by the Research Ethics Committee of the FURB (2.969.842).

## Results

The study population consisted of 2488 participants. Of these, 235 (9.4%) did not go to the Examination Center and 300 (12.1%) refused to perform the carotid ultrasound. We analyzed 1953 participants.

Table 1 shows the characteristics of the participants. The mean age was 50.85 years (SD=14.64) and median was 51.4 years old. There was a higher prevalence of female participants, married, white and Germanic. Regarding the level of education, most participants have completed between one and four years of primary education. Economic class B1/B2 predominated. As for lifestyle, low alcohol consumption was observed in the study population, as well as a high percentage of people who had never smoked, and a low percentage of sedentary people. With regard to anthropometric data, there was a high prevalence of obesity, high-risk WC, high-risk WHR and high-risk WtHR. It was also possible to observe a lower percentage of participants with chronic diseases: hypertension, diabetes and dyslipidemia, when compared to participants without chronic diseases. In this study, the presence of carotid plaques was observed in 36% of participants: 64.0% had no plaques, 23.3% had one or two plaques and 12.8% had three or more carotid plaques.

**Table 1:** – Characteristics of the SHIP- Brazil study participants 2014-2018

Variables		Population characteristics (n = 1953)	
		Frequency	Percentage
**1 – Sex**	Male	820	42.0%
	Female	1133	58.0%
**2 – Age group**	20-39	498	25.5%
	40-59	885	45.3%
	60-79	570	29.2%
**3 – Marital status**	Married	1482	77.2%
	Single/Separated/Widowed	438	22.8%
**4 – Race/Color**	White	1779	92.8%
	Non-white	138	7.2%
**5 – Germanic culture**	No	572	29.8%
	Yes	1345	70.2%
**6 – Education**	Higher Education	263	13.9%
	High School	493	26.0%
	Elementary School (5-8 grade)	374	19.8%
	Elementary School (1-4 grade)	739	39.0%
	Illiterate	24	1.3%
**7 – Economic Class**	A1/A2	244	12.5%
	B1/B2	1148	58.8%
	C1/C2	547	28.0%
	D/E	13	0.7%
**8 – Alcohol consumption**	Low	1346	70.8%
	Moderate	322	16.9%
	High	150	7.9%
	Severe	83	4.4%
**9 – Smoking**	Never smoked	1271	66.7%
	Smoker	434	22.8%
	Former smoker	202	10.6%
**10 – Physical Activity**	Active	1239	68.3%
	Sedentary	575	31.7%
**11 – Obesity**	Low weight	15	0.8%
	Eutrophic	445	24.2%
	Overweight	668	36.3%
	Obese	712	38.7%
**12 – Waist Circunference**	Low risk	632	34.6%
	High risk	1194	65.4%
**13 – Waist-to-height ratio**	Low risk	558	30.5%
	High risk	1269	69.5%
**14 – Waist-to-hip ratio**	Low risk	551	30.1%
	High risk	1277	69.9%
**15 – Hypertension**	No	1100	63.2%
	Yes	640	36.8%
**16 – Diabetes**	No	1719	91.0%
	Yes	171	9.0%
**17 – Dyslipidemia**	No	1297	68.2%
	Yes	604	31.8%
**18 – Carotid plaques**	0	1249	64.0%
	1-2	455	23.3%
	≥3	249	12.8%

Source: The Study of Health in Pomerania (SHIP) – Brazil

Tables 2 and 3 show the association between the variables presented and the presence of atherosclerotic plaques in the carotid artery. There was a higher percentage of participants without carotid plaques with the following characteristics: female gender, age range between 20 and 39 years, single/separated/widowed, non-white, non-Germanic culture, complete higher education, economic class B1/B2, who drink moderate amounts of alcohol, who have never smoked, and who practiced physical activity. As for the anthropometric data, the highest percentage of people without plaques was in the eutrophic group, with low-risk WC measurement, low-risk WHR and low-risk WtHR measurements. Regarding chronic diseases, there was a higher percentage of individuals without plaques in non- hypertensive, non-dyslipidemic and non-diabetic ones.

**Table 2:** – Association between sociodemographic and lifestyle variables and cardiovascular risk with the presence of carotid plaques in participants in the SHIP-Brazil study 2014-2018

Characteristics		Plaque score			p-value
		0 n = 1249	1 ou 2 n = 455	≥3 n = 249	
**1 – Marital status**	Married	948 (64%)	350 (23.6%)	184 (12.4%)	0.690
	Single / Divorced / Widowed	290 (66.2%)	97 (22.2%)	51 (11.6%)	
**2 – Germanic culture**	No	458 (80.1%)	84 (14.7%)	30 (5.2%)	<0.001
	Yes	778 (57.8%)	362 (26.9%)	205 (15.2%)	
**3 – Education**	Higher Education	231 (87.8%)	25 (9.5%)	7 (2.7%)	<0.001
	High School	407 (82.6%)	70 (14.2%)	16 (13.7%)	
	Elementary School (5 - 8 grade)	261 (69.8%)	77 (20.6%)	36 (9.6%)	
	Elementary School (1 - 4 grade)	310 (42.0%)	252 (34.1%)	177 (24.0%)	
	Illiterate	10 (41.7%)	11 (45.8%)	3 (12.5%)	
**4 – Economic Class**	A1/A2	137 (56.2%)	66 (27.0%)	41 (16.8%)	<0.001
	B1/B2	784 (68.3%)	251 (21.9%)	113 (9.8%)	
	C1/C2	320 (58.5%)	136 (24.9%)	91 (16.6%)	
	D/E	8 (61.5%)	1 (7.7%)	4 (30.8%)	
**5 – Alcohol consumption**	Low	828 (61.5%)	337 (25.0%)	181 (13.5%)	0.001
	Moderate	239 (74.2%)	57 (17.7%)	26 (8.1%)	
	High	107 (71.3%)	28 (18.7%)	15 (10.0%)	
	Severe	55 (66.3%)	18 (21.7%)	10 (12.0%)	
**6 – Smoking**	Never smoked	903 (71.0%)	263 (20.7%)	105 (8.3%)	<0.001
	Smoker	235 (54.2%)	118 (27.2%)	81 (18.7%)	
	Former smoker	97 (48.0%)	59 (29.2%)	46 (22.8%)	
**7 – Physical Activity**	Active	829 (66.9%)	263 (21.1%)	147 (11.9%)	0.014
	Sedentary	348 (60.5%)	156 (27.1%)	71 (12.4%)	

Source: The Study of Health in Pomerania (SHIP) – Brazil

**Table 3:** – Association between clinical and anthropometric variables and cardiovascular risk with the presence of carotid plaques in participants in the SHIP-Brazil study 2014-2018

Characteristics		Plaques score			Valor p
		0 n = 1249	1 ou 2 n = 455	≥3 n = 249	
**1 – Sex**	Male	481 (58.7%)	199 (24.3%)	140 (17%)	<0.001
	Female	768 (67.8%)	256 (22.6%)	109 (9.6%)	
**2 – Age group**	20-39	483 (97%)	14 (2.8%)	1 (0.2%)	<0.001
	40-59	623 (70.4%)	206 (23.3%)	56 (6.3%)	
	60-79	143 (25.1%)	235 (41.2%)	192 (33.7%)	
**3 – Race / Color**	White	1126 (63.3%)	426 (24%)	227 (12.8%)	<0.001
	Non-white	111 (80.4%)	21 (15.2%)	6 (4.4%)	
**4 – Obesity**	Underweight	11 (73.3%)	0 (0%)	4 (26.7%)	<0.001
	Eutrophic	329 (73.9%)	68 (15.3%)	48 (10.8%)	
	Overweight	442 (66.2%)	151 (22.6%)	75 (11.2%)	
	Obese	401 (56.3%)	209 (29.4%)	102 (14.3%)	
**5 – Abdominal circumference**	Low risk	465 (73.6%)	99 (15.7%)	68 (10.8%)	<0.001
	High risk	713 (59.7%)	326 (27.3%)	155 (13.0%)	
**6 – Waist-to-height ratio**	Low risk	424 (76.0%)	79 (14.2%)	55 (9.9%)	<0.001
	High risk	754 (59.4%)	347 (27.3%)	168 (13.2%)	
**7 – Waist-to-hip ratio**	Low risk	467 (84.8%)	57 (10.3%)	27 (4.9%)	<0.001
	High risk	712 (55.8%)	369 (28.9%)	196 (15.4%)	
**8 – Hypertension**	No	845 (76.8%)	188 (17.1%)	67 (6.1%)	<0.001
	Yes	279 (43.6%)	216 (33.8%)	145 (22.7%)	
**9 – Diabetes**	No	1160 (67.5%)	381 (22.2%)	178 (10.4%)	<0.001
	Yes	65 (38.0%)	56 (32.8%)	50 (29.2%)	
**10 – Dyslipidemia**	No	923 (71.2%)	256 (19.7%)	118 (9.1%)	<0.001
	Yes	303 (50.2%)	185 (30.6%)	116 (19.2%)	

Source: The Study of Health in Pomerania (SHIP) – Brazil

A higher percentage of participants with one or two atherosclerotic plaques were found with the following characteristics: male, aged between 60 and 79, married, white, Germanic culture, illiterate, economic class A1/A2, low alcohol consumption, smokers, insufficiently active, hypertensive, diabetic and dyslipidemic. As for anthropometric data, there was a higher percentage of obesity, high-risk WC and high-risk WtHR measurements, and low-risk WHR.

There was a higher percentage of participants with three or more plaques among men; age group from 60 to 79 years old; married; whites; Germanic culture; education with 1-4 complete years of primary education; economy class A1/A2; low alcohol consumption; smokers, sedentary, hypertensive, diabetic and dyslipidemic. Regarding anthropometric data, underweight, waist circumference with high-risk measurements, waist-height ratio with high-risk measurements and waist-hip ratio with high-risk measures.

With regard to the presence or absence of plaques, there was a higher prevalence of plaques in men, aged 60-79, married people, white people, Germanic culture, illiterate people, economic class A1/A2, participants with low alcohol consumption, smokers, sedentary participants, obese, hypertensive, diabetic and dyslipidemic participants.

## Discussion

Carotid atherosclerosis has been described in a considerable portion of populations in Latin America for decades. A Mexican study published in 1999 reported a prevalence of atherosclerotic carotid lesion in 64.8% of the studied population.^18^ A Brazilian study^19^ published in 2021 demonstrated presence of plaques in 35.8% of the population.

About gender, we found that the prevalence of people with carotid plaques was higher among men. This was observed both in participants with one or two plaques and in those with three or more plaques. This information is in line with the findings of previous studies^8,20,21^that demonstrated that men are at greater risk of cardiovascular events. On the other hand, in women after the menopause, this risk is equalized by the lack of the protective effect that estrogen provides.^22-24^

It was also observed that as the age group increased, there was an increase in the prevalence of participants with carotid plaques. Noteworthy, the percentage of participants with carotid plaques in the 40-59 age group was significantly higher than the percentage found in the 20-39 age group. Also, the percentage of participants with carotid plaques in the 60-79 age group rose from 29.6% to 74.9% when compared to the previous age group. This characteristic was found in other studies, which observed a substantial increase in plaques in participants aged 50 and over.^20,21,25,26^

The presence of hypertension is an important risk factor for the development of atherosclerotic plaques, as has been shown in various studies.^8,21,22,25-30^ In the same way, diabetes is characterized in various literature sources as an important comorbidity associated with plaque formation.^20,21,25,26^

In our study, the presence of dyslipidemia was also found to be highly correlated with the presence of plaques. In the dyslipidemic group, atherosclerotic plaques were present in more than half of the participants. This correlation was also observed in several studies in which this variable was analyzed.^21,25,26^

With regard to lifestyle, sedentary people had a higher prevalence of carotid plaques than physically active people. This finding was statistically significant (p=0.014), and this association has also been observed in other studies.^21,22,25,26^With regard to smoking, a higher percentage of plaques was found in current smokers, but there was also a high prevalence of plaques in former smokers. Studies suggest that the rate of atherosclerotic change can be reduced by smoking cessation, but a residual effect seems to be present for at least 10 years.^31,32^The chronology of these facts could not be observed in our study, as it was a cross-sectional study. This factor can be explained by the chronic inflammation process associated with smoking, which can damage the endothelial cells of the carotid arteries and favor the accumulation of thrombogenic factors.^33^

As for alcohol use, there was a higher prevalence of plaques in people with low alcohol consumption. Another study based on the SHIP used the carotid intima-media layer as a method of assessing subclinical atherosclerosis and found an inversely proportional association between alcohol consumption and an increase in the thickness of the carotid intima-media layer, another subclinical marker of atherosclerosis. This association was observed in men with a daily intake of up to 80g of alcohol.^34^

Our results demonstrate an association between obesity and the presence of plaques. A similar relationship was also observed between high-risk measures of waist circumference, waist-to-height ratio and waist- to-hip ratio and the presence of plaques. This association has been found in several other previously published studies.^21,25,26^

With regard to schooling, lower levels were associated with higher prevalence of plaques. This relationship can be explained by the low level of education of this population in relation to disease prevention. For economic class, the highest percentage of participants with 1 or 2 plaques was in the A1/A2 economic class population (27.1%), and the highest prevalence of 3 or more plaques was in the D/E class population (30.8%). Studies such as those carried out previously have associated the participant’s low level of education and social class with the presence of subclinical atherosclerosis in the participants.^26^Other studies can demonstrate whether individuals from higher social classes may have better conditions to access complementary exams, while lower social classes would only reach the diagnosis when the disease is more advanced.

As for ethnicity, there was a higher prevalence of carotid plaques in white people. According to the American Heart Association’s prevention guideline, the estimated risk of developing atherosclerotic cardiovascular disease within 10 years is higher in people of African- American descent.^34^We observed a higher prevalence of plaques in participants of Germanic culture (assumed here as *proxy* of German descendent). A study evaluating Germanic descendants in Vale do Itajaí region showed that first-generation Germanic descendants had no significant differences in terms of cardiovascular risk factors when compared to German immigrants, except for HDL cholesterol, which was higher in the Germanic population, but this lipid fraction was not analyzed in our population.^35^

There are no data in the literature that indicate significant differences in the number of atherosclerotic plaques. Similarly, we did not observe any significant differences between the groups with 1 to 2 plaques and those with 3 or more carotid plaques that could influence changes in clinical practice with regard to this information.

The importance of studying risk factors and prevalent characteristics in association with atherosclerotic disease can lead to prevention and control measures to combat its harmful effects. But it is important to mention that this study has certain limitations. As it is a sectional study, it does not define a cause-and-effect relationship between the variables, only raising hypotheses that require new and more in-depth analyses in longitudinal studies to confirm them. Also noteworthy is the fact that some clinical information is self-reported by the population under analysis, and the high number of losses and refusals, which may generate bias. This factor can be better assessed in the future through records in medical records and by carrying out additional confirmatory tests.

## Conclusions

The conclusion of our study, as seen in the central figure, was that there was an association between the presence of cardiovascular risk factors and the presence of carotid plaques in the participants. Plaques were more prevalent in hypertensive, dyslipidemic, diabetic, smoking, sedentary and obese individuals. As for the characteristics of the population, plaques were more prevalent in: men, aged between 60 and 79, white, Germanic culture, illiterate, economic class A1/A2, and low alcohol consumption. Atherosclerotic plaques were more prevalent in participants who had high-risk measurements of waist circumference, waist-to-hip ratio and waist-to-height ratio.

These findings highlight the importance of identifying risk groups that would benefit more consistently from cost-effective screening protocols. These protocols should identify patients who would benefit from morbidity and mortality, without being exposed to unnecessary invasive procedures that can lead to physical and psychological harm.

Thus, longitudinal studies and experimental designs are needed to improve the assessment of causality and risk progression.
